# Sustainable Synthesis of NiMo Alloy Nanoparticles for Hydrogen Evolution Catalysis Using a Recyclable Ionic Liquid Solvent

**DOI:** 10.1002/cssc.70620

**Published:** 2026-04-13

**Authors:** Allison P. Forsberg, Yashna Khakre, Smaranda C. Marinescu, Richard L. Brutchey

**Affiliations:** ^1^ Department of Chemistry University of Southern California Los Angeles California USA

**Keywords:** bimetallic alloy, hydrogen evolution, ionic liquid, microwave synthesis, recyclable solvent

## Abstract

Advancing the sustainability of nanoparticle electrocatalyst synthesis requires reducing solvent waste without compromising catalytic performance. Here, we report a rapid, microwave‐assisted colloidal synthesis of NiMo alloy nanoparticles in the ionic liquid 1‐butyl‐3‐methylimidazolium bis(trifluoromethylsulfonyl)imide (BMIM‐NTf_2_), which serves as both reaction medium and recyclable solvent. NiMo nanoparticles with tunable compositions were obtained within 1 min at 230°C, with Ni_0.86_Mo_0.14_ giving the highest activity toward the hydrogen evolution reaction (HER), achieving an onset potential of −129 mV, an overpotential at 10 mA cm^−2^ of −290 mV versus RHE in acidic electrolyte, and >99% Faradaic efficiency. The ionic liquid was recovered and purified via aqueous NH_4_OH extraction, removing over 99% of residual Mo species, and reused for up to five successive syntheses. Nanoparticles prepared in recycled BMIM‐NTf_2_ retained comparable crystallinity, morphology, and catalytic activity, with stable HER performance over 45 h of electrolysis. These results demonstrate that ionic liquid recycling can improve the sustainability of the preparation of NiMo nanoparticle electrocatalysts while preserving performance and material integrity.

## Graphical abstract

NiMo alloy nanoparticles synthesized in BMIM‐NTf_2_ ionic liquid exhibit composition‐dependent hydrogen evolution activity. The ionic liquid solvent is recovered, purified, and reused for six synthesis cycles while preserving nanoparticle structure and catalytic performance. A recyclable ionic liquid platform thus enables sustainable production of hydrogen evolution reaction‐active NiMo nanoparticles with reduced organic solvent waste.

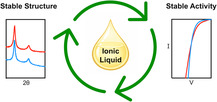

## 1 Introduction

Nanoparticle syntheses are often conducted in high boiling hydrocarbon solvents such as octadecene, oleylamine, or squalane [1, 2], which can tolerate the elevated temperatures required for colloidal nucleation and growth. However, these hydrocarbon solvents are still considered volatile organic compounds (VOCs) that pose environmental health and safety risks due to their flammability, toxicity, and contribution to air pollution. In addition, they are typically discarded after a single use, generating waste and increasing processing costs [3]. Efforts to mitigate these issues have led to the exploration of more sustainable solvent systems that can reduce emissions, improve safety, and enable recyclability. Among these alternatives, ionic liquids (ILs) have emerged as promising candidates. ILs are molten salts composed of organic cations and organic or inorganic anions that are liquid below 100°C. Their negligible vapor pressures, nonflammability, and high thermal and chemical stability improve process safety and reduce fugitive emissions relative to conventional VOC solvent media [4]. The vast structural diversity of ILs allows their physicochemical properties, such as viscosity and hydrophobicity, to be tuned to meet the requirements of specific reactions, making them attractive as functional and tunable reaction solvents [5]. Moreover, their high polarity and negligible vapor pressures enable efficient microwave absorption, making ILs particularly effective for microwave‐assisted reactions which often require less thermal input than traditional batch reactions [6, 7]. Most importantly, the tunable hydrophobic or hydrophilic character of ILs enables efficient separation from reaction products when used as solvent media, facilitating recovery and reuse [8, 9]. In contrast to conventional organic solvents, which are typically discarded after a single use, ILs can be recycled over multiple cycles while maintaining their molecular integrity [10, 11]. The use of IL solvents in colloidal nanoparticle syntheses offers additional advantages arising from their unique interfacial and solvation characteristics [12, 13]. Their low interfacial tension promotes rapid nucleation and growth, while their high dielectric constants and ionicity stabilize nanoparticles electrostatically, suppressing aggregation and Ostwald ripening [14]. These characteristics of IL solvents enable the synthesis of small, well‐defined nanoparticles, providing a versatile platform for studying functional nanoparticles.

Rapid nucleation and growth can kinetically trap atoms into metastable arrangements at the nanoscale, a strategy that is particularly important for metallic systems such as Ni–Mo, which are largely immiscible under equilibrium conditions [7, 15]. The substantial atomic size mismatch between Ni (atomic radius = 124 pm) and Mo (atomic radius = 139 pm) increases lattice strain when attempting to form an alloy, and the differing parent crystal structures of Ni (FCC) and Mo (BCC) at low temperatures make continuous alloy formation energetically unfavorable. At the nanoscale, however, or via nonequilibrium techniques such as electrodeposition or rapid quenching, Mo atoms can be incorporated into an FCC Ni crystal lattice, forming a metastable FCC NiMo alloy.

Beyond their structural interest, these NiMo alloy nanoparticles have proven highly effective as electrocatalysts for the hydrogen evolution reaction (HER) [16, 17]. Hydrogen is an attractive alternative to fossil fuels due to its high gravimetric energy density and the absence of carbon emissions at the point of use in fuel cells [18]. Although Pt/C remains the industrial benchmark for electrochemical water reduction, the need for efficient HER catalysts based on earth‐abundant and inexpensive metals is increasingly important [19]. Several earth‐abundant bimetallic catalysts have shown promising HER activity in acidic media. For example, NiFe alloy nanoclusters exhibited an overpotential of −203 mV at 10 mA cm^−2^ [20], while Ni_*x*_W_1–*x*_ alloy nanoparticles achieved overpotentials as low as −143 mV, with Ni_0.7_W_0.3_ exhibiting the best performance [21]. NiMo alloy nanoparticles have demonstrated particularly strong HER activity in both acidic and alkaline media [17, 22]. Gray et al. reported NiMo nanopowders with overpotentials of −70 and −80 mV at 20 mA cm^−2^ in alkaline and acidic media, respectively, though with limited stability [16]. More recently, Zheng and Li showed that N‐doped graphite nanotube‐supported NiMo catalysts achieved an overpotential of −65 mV at 10 mA cm^−2^ in acidic media with stability up to 15 h [17]. The compositional tunability of NiMo alloys enables modulation of their electronic structure, thereby optimizing hydrogen adsorption energetics and HER kinetics [23].

While these catalytic advances are significant, the sustainability of the synthetic process itself remains an important consideration. Although NiMo alloy nanoparticles can be synthesized using conventional solvents, achieving a more environmentally sustainable process requires careful consideration of solvent use. IL solvents, with their negligible vapor pressures, high stability, and tunable physicochemical properties, offer a promising solution for mitigating solvent waste. However, it is important to recognize that ILs are not inherently ‘green.’ Previous life cycle assessments of imidazolium‐based ILs have shown that their upstream production can carry substantial environmental burdens, in some cases exceeding those of conventional VOC solvents when used in a once‐through manner. These studies underscore that environmental competitiveness depends critically on effective recovery and reuse [24, 25]. In parallel, techno‐economic analyses of BMIM‐NTf_2_ systems have demonstrated that the high purchase cost of ILs can render them economically impractical relative to conventional VOC solvents when used once, but that solvent recycling can restore cost competitiveness [10, 11]. Building on these insights, herein we report the synthesis of NiMo nanoparticle electrocatalysts using 1‐butyl‐3‐methylimidazolium bis(trifluoromethylsulfonyl)imide (BMIM‐NTf_2_) IL as the reaction solvent, alongside the successful recycling and reuse of the IL. The primary focus of this work lies in prioritizing the lifecycle of the reaction media over incremental gains in catalytic metrics. As proof of concept, the NiMo alloy nanoparticles synthesized in both pristine and recycled IL exhibit comparable electrochemical HER activity and stability under acidic conditions for up to 45 h, demonstrating that the IL can be continuously recycled and reused without compromising electrocatalytic performance. We further show that the catalyst features required for efficient HER are preserved, offering a closed‐loop, atom‐economical alternative to traditional synthesis of catalytically active alloy nanoparticles.

## 2 Experimental Section

Ni(acetylacetonate)_2_ (Ni(acac)_2_, 95%; Sigma–Aldrich), Mo(CO)_6_ (98%; Sigma–Aldrich), aqueous NH_4_OH (14.8 M; Sigma–Aldrich), dichloromethane (DCM; Fisher Scientific) were all used as received. 1‐butyl‐3‐methylimidazolium bis(trifluoromethylsulfonyl)imide (BMIM‐NTf_2_; IOLITEC Lot: W006x106.2.1‐IL0029, clear light yellow) was dried under vacuum at 120°C for 2 h prior to use and stored under N_2_. Oleylamine (70% Sigma–Aldrich) and 1‐octadecene (90% Sigma–Aldrich) were dried under vacuum at 120°C for 2 h prior to use and stored under N_2_.

### 2.1 Nanoparticle Synthesis

NiMo alloy nanoparticles were synthesized with compositional Ni:Mo feed ratios of 4:1, 1:1, 1:4, 1:8, and 1:10 at a fixed concentration. As a representative procedure for the 1:1 composition, Ni(acac)_2_ (0.103 g, 0.400 mmol) and Mo(CO)_6_ (0.106 g, 0.400 mmol) were combined in an oven‐dried Biotage Initiator+ microwave vial (2–5 mL size) with 2.5 mL (3.6 g) of BMIM‐NTf_2_. The microwave vial was sealed and then sparged with N_2_ for 30 min. The vial was then placed in the Biotage Initiator+ microwave and heated to 230°C for 1 min; power was controlled by the microwave with full power (300 W) output until the temperature was reached, and then the power was reduced as determined by the system to maintain the set reaction temperature. The solution went from a clear yellow metal salt solution to a black colloidal suspension. In the workup procedure, the nanoparticle suspension was split between two centrifuge tubes and washed with acetone (∼40 mL) followed by vortex mixing and bath sonication (5 min) before precipitation of the nanoparticles via centrifugation (6000 rpm, 5 min). After centrifugation, the supernatant, which contains the IL, was saved in a round‐bottom flask and the nanoparticles were resuspended in a minimal volume of acetone and bath sonicated (1 min) and vortex mixed. To precipitate the nanoparticles, hexanes (∼35 mL) was added, and the mixture was bath sonicated and vortex mixed for 5 min and centrifuged again (6000 rpm, 5 min). The clear and colorless supernatant was discarded, and the resulting nanoparticles were dried or resuspended in acetone for further characterization. As a comparison, the 1:1 alloy was also synthesized in organic solvent as follows: Ni(acac)_2_ (0.103 g, 0.400 mmol) and Mo(CO)_6_ (0.106 g, 0.400 mmol) were combined with oleylamine (0.5 mL) and 1‐octadecene (2 mL) in an oven dried Biotage Initiator+ microwave vial (2–5 mL size). The microwave vial was sealed and then sparged with N_2_ for 30 min. The vial was placed in the Biotage Initiator+ microwave and heated following the same procedure as detailed above. The solution went from a cloudy blue metal salt solution to a black colloidal suspension. The nanoparticle suspension was split between two centrifuge tubes and washed with minimal hexanes (∼5 mL) to facilitate transfer of the reaction suspension from the microwave vial into the centrifuge tubes. Ethanol (∼40 mL) was added to each centrifuge tube followed by vortex mixing and bath sonication before centrifugation (6000 rpm, 5 min). The supernatant was collected, and the high‐boiling organic solvents were concentrated to yield a pink/brown liquid.

### 2.2 IL Recycling

The supernatant obtained from the previous step, consisting primarily of IL and acetone, was concentrated by rotary evaporation, leaving behind a dark brown IL phase. To this residue, approximately 10 mL of aqueous NH_4_OH, 2 mL of water, and 3 mL of dichloromethane (DCM) were added to facilitate extraction of unreacted Mo salts from the IL. The mixture was vigorously stirred for 2 h, then transferred to a separatory funnel. The original flask was washed with additional DCM to recover as much IL as possible, and the combined solution was shaken and allowed to separate. The IL/DCM layer was collected, dried over Na_2_SO_4_, washed with DCM, and finally concentrated to yield a clear yellow IL solution.

### 2.3 Synthesis of NiMo Alloy Nanoparticles from Recycled IL (NiMo‐R)

The freshly recycled IL was weighed into a new microwave vial, and additional virgin IL was added to bring the total solvent mass to that used in the initial reaction (3.6 g BMIM‐NTf_2_). Ni(acac)_2_ (0.103 g, 0.4 mmol) and Mo(CO)_6_ (0.106 g, 0.4 mmol) were then added. The reaction was carried out following the procedure described in the nanoparticle synthesis above to yield an identically colored black colloidal suspension.

### 2.4 Characterization

Powder X‐ray diffraction (PXRD) was carried out on a Rigaku Miniflex diffractometer using Cu *K*α radiation (*λ* = 1.541 Å) in a *θ–*2*θ* reflection geometry under ambient conditions. Samples were resuspended in acetone and drop cast on a zero‐diffraction silicon substrate. Scanning electron microscopy coupled with energy dispersive X‐ray spectroscopy (SEM‐EDX) was carried out on nanoparticle samples resuspended in minimal acetone and drop cast onto a clean piece of copper (1 cm^2^) and imaged using a Thermo Scientific Apreo 2 SEM using an ETD detector and an operating voltage of 20 kV with an Oxford UltimMax 170 Silicon Drift EDX Detector. EDX quantification was carried out using Aztec software from Oxford Instruments. An example elemental quantification graph is provided for alloy B (Figure S1). Transmission electron microscopy (TEM) was carried out on a Thermo Scientific FEI Talos F200C G1 microscope with an operating voltage of 200 kV. Scanning TEM (STEM) coupled with EDX was carried out on the FEI Talos F200 as well using both a high angle annular dark field (HAADF) detector and an Oxford X‐MaxN 100 TLE Windowless SSD. Samples were drop cast on holey carbon on copper grids (Ted Pella, Inc.). To help with separation of the nanoparticles for TEM imaging, 500 μL of oleylamine was added prior to workup and then probe sonicated for 5 min in ice using a Sonics Vibracell probe sonicator. Fourier transform infra‐red (FT‐IR) spectra were acquired on a Bruker Vertex FTIR 80V spectrometer under vacuum (*P *= 4.2 hPa). Nanoparticles were dried and mixed with oven‐dried KBr (4 mg in a 200 mg KBr matrix) and pressed in a pellet press. The FT‐IR spectrum for BMIM‐NTf_2_ was acquired by diluting BMIM‐NTf_2_ in dichloromethane and dropping one drop onto an NaCl plate. Solution ^1^H NMR spectra were collected using a Varian 500 MHz NMR spectrometer in CDCl_3_ and processed using MestreNov software. Inductively coupled plasma‐optical emission spectroscopy (ICP‐OES) was carried out by Galbraith laboratories (Knoxville, TN) testing for Mo and Ni. A mass of 289.52 mg of BMIM‐NTf_2_ was used for each metal analysis. X‐ray photoelectron spectroscopy (XPS) was performed on ThermoFisher ESCALAB‐QXi instrument, with the analysis chamber pressure at 10^−7^ Torr. An Al *K*α source was used. Pass energies of 160 and 20 eV were used for acquiring the survey and high‐resolution spectra, respectively.

### 2.5 Preparation of Working Electrode

Ti foil (99.99%, 0.25 mm thick) was cut into 1 cm × 4 cm pieces. The active area for deposition (0.5 cm × 1 cm) on the lower half of each Ti piece was defined using a silicone grease mask to confine the drop cast suspension. An acetone suspension of NiMo nanoparticles was drop‐cast onto the defined area to achieve a loading of 0.5 mg cm^−2^. After deposition, the grease mask was removed, and the nanoparticle‐modified Ti foils were annealed for 1 h at 450°C under a flow of 5% H_2_/N_2_. The electrodes were then transferred to a N_2_‐filled glove bag, and all areas of the Ti foil outside the nanoparticle active region were coated with a two‐part epoxy (J‐B Weld ClearWeld) and allowed to cure. The electrodes were stored in a N_2_‐filled glovebox until electrochemical measurements were performed. The geometric active surface area of each electrode was remeasured by light microscopy using ImageJ software.

### 2.6 Electrochemical Measurements

The hydrogen evolving activity of the NiMo alloy nanoparticles was evaluated electrochemically. Measurements were conducted in a custom‐built H‐cell in which the working and the counter compartments were separated by a fine‐porosity glass frit. A VersaSTAT 3 potentiostat (Princeton Applied Research) was used. All experiments employed a three‐electrode configuration, with the modified Ti electrodes as the working electrode, a graphite rod as the counter electrode, and a 1 M KCl‐saturated Ag/AgCl electrode as the reference electrode. The electrolyte was a 0.1 M NaClO_4_ aqueous solution (pH 1.3), adjusted by the addition of concentrated H_2_SO_4_ (18.7 M). Linear sweep voltammetry (LSV) was performed at a scan rate of 10 mV s^−1^. The polarization curves were manually corrected for internal solution resistance (*R*_s_) according to the equation: *E*_corrected_ = *E*_measured_ – *i* * *R*_s_, where *E*_corrected_ is the corrected voltage, *E*_measured_ is the applied voltage, *i* is the raw measurement current, and *R*_s_ is the solution resistance estimated from electrochemical impedance spectroscopy (EIS) using a three‐time‐constant (3TS) model [26]. The *E*_corrected_ was referenced to RHE by adding (0.205 + 0.059 × pH) V. EIS measurements were performed to obtain the solution resistance near the open circuit potential (OCP), over the frequency range 100 kHz – 0.1 Hz using a 10 mV sinusoidal perturbation. Double layer capacitance (*C*_dl_) was measured via cyclic voltammetry (CV) experiments carried out near the OCP region in a 100 mV window (−50 to 50 mV vs. Ag/AgCl), at scan rates of 10, 20, 30, 40, 50, and 60 mV s^−1^. The absolute difference in the cathodic and anodic current values (*i*_c_–*i*_a_) obtained at 0 mV vs. Ag/AgCl were plotted vs. the scan rate (V s^−1^), and the *C*_dl_ values were obtained as half of the slope of the linear fit. ECSA was obtained via the equation ECSA = *C*_dl_/*C*_s_, where *C*_s_ is 0.035 mF cm^−2^ [27]. Gas chromatography (GC) analysis and quantification of gaseous products during the electrochemical experiments was carried out using a Shimadzu Nexis‐II GC 2030 equipped with a thermal conductivity detector and argon as carrier gas.

## 3 Results and Discussion

### 3.1 NiMo Nanoparticle Synthesis

Colloidal syntheses of NiMo alloy nanoparticles have traditionally relied on high‐boiling organic solvents, such as oleylamine and octadecene [23]. These reactions typically require extended heating (e.g., 320°C for 2 h) and generate solvent waste, as the organic media are typically not recycled, contributing to both environmental impact and cost [10]. In this work, the IL BMIM‐NTf_2_ was employed as the sole reaction solvent. BMIM‐NTf_2_ was selected for its high thermal stability (*T*_dec_ = 369°C) [28], negligible vapor pressure, and ability to solubilize both metal precursors. The Ni(acac)_2_ and Mo(CO)_6_ precursors were combined in varying feed ratios to produce five alloy compositions (*A–* *E*), corresponding to nominal Ni:Mo ratios of 4:1, 1:1, 1:4, 1:8, and 1:10, respectively. After 1 min of microwave heating at 230°C, the resulting NiMo alloy nanoparticles were precipitated and isolated, while the IL solvent was recovered for recycling (vide infra). Control experiments revealed that Mo(CO)_6_ alone yielded nanoparticles, whereas Ni(acac)_2_ alone did not, suggesting that zero‐valent Mo likely mediates the reduction of Ni^2+^ during the alloying process, with oxidized Mo species likely remaining dissolved in the IL. The isolated NiMo alloy nanoparticles were magnetic, consistent with the presence of zero‐valent Ni^0^.

**FIGURE 1:**
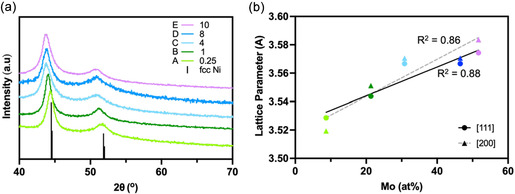
(a) Powder XRD patterns for NiMo alloy samples *A–* *E*. As the amount of incorporated Mo increases, the diffraction pattern shifts to a lower 2*θ* consistent with Vegard's law. (b) Plot of lattice parameter *a* versus the atomic percent of Mo incorporated into alloys *A–* *E*, as measured by XRD and SEM‐EDX, respectively.

PXRD confirmed that the five NiMo nanoparticle compositions adopt the FCC structure of Ni (Figure 1a), with no evidence of secondary crystalline phases. As the nominal Mo content increased, the resulting NiMo alloy nanoparticles exhibit a gradual shift of the diffraction peaks to lower 2*θ* values, consistent with lattice expansion arising from substitution of larger Mo atoms into the Ni lattice. A plot of the lattice parameter (*a*) versus the experimentally determined alloy compositions produced a reasonably linear correlation consistent with Vegard's law, suggesting the formation of alloys (Figure 1b). The experimentally determined elemental compositions of the nanoparticles were determined by SEM‐EDX. A linear regression of the experimentally measured Ni:Mo ratios versus the nominal feed ratios yielded a slope of 4 (Figure S2), indicating that only ca. 25% of the added Mo was incorporated into the alloy phase. Nevertheless, alloy formation was achieved across all compositions, yielding nanoparticles of the formula Ni_0.91_Mo_0.09_ (*A*), Ni_0.86_Mo_0.14_ (*B*), Ni_0.69_Mo_0.31_ (*C*), Ni_0.53_Mo_0.47_ (*D*), and Ni_0.48_Mo_0.52_ (*E*).

**FIGURE 2:**
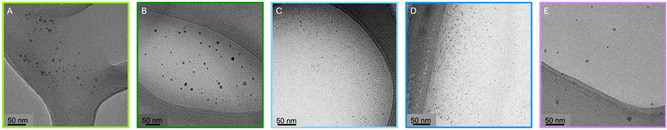
Transmission electron micrographs of alloys *A–* *E*. The scale bar represents 50 nm.

TEM revealed that the NiMo alloy nanoparticles are similarly sized and shaped, possessing quasi‐spherical morphologies and average diameters ranging from 2.3 ± 0.5 to 6.4 ± 1.5 nm across the compositional series (Figures 2 and S3). High‐angle annular dark‐field scanning TEM (HAADF–STEM) coupled with EDX elemental mapping of representative composition *B* further confirmed the homogeneous distribution of Ni and Mo throughout individual particles, consistent with alloy formation rather than phase‐segregated or multidomain structures (Figure S4).

FT‐IR spectroscopy was used to determine whether the IL remained associated with the NiMo nanoparticle surfaces after workup. Characteristic bands of the NTf_2_^−^ anion were observed at 1052 and 1345 cm^−1^ for the symmetric and asymmetric *v*(SO_2_) stretching, along with a *v*(C—F) stretching band at 1183 cm^−1^. A *v*(C—N) stretching band corresponding to the imidazolium ring was observed at 1560 cm^−1^ (Figure S5) [29]. The presence of these features indicates that BMIM‐NTf_2_ remains adsorbed on the nanoparticle surfaces after isolation. This surface interaction is consistent with the strong ionic interactions between the IL and the nanoparticle surface.

### 3.2 Electrochemical Studies

Working electrodes were prepared from each of the five NiMo alloys (*A–* *E*) by drop‐casting an acetone suspension of the nanoparticles onto Ti foil substrates to achieve a mass loading of 0.5 mg cm^−2^. The electrodes were subsequently annealed under forming gas (450°C, 1 h) to remove residual IL and surface oxides [30, 31]. SEM‐EDX performed before and after annealing confirmed that the treatment did not alter either the elemental distribution or the overall Ni:Mo ratio within the alloy nanoparticles (Figure S6 and Table S1). The HER activity of the NiMo alloys was then evaluated in a three‐electrode configuration, employing the annealed Ti‐supported nanoparticles as the working electrodes, a graphite rod as the counter electrode, and a 1 M KCl‐saturated Ag/AgCl electrode as the reference. Electrochemical measurements were conducted in 0.1 M NaClO_4_ (pH 1.3) aqueous electrolyte.

**FIGURE 3:**
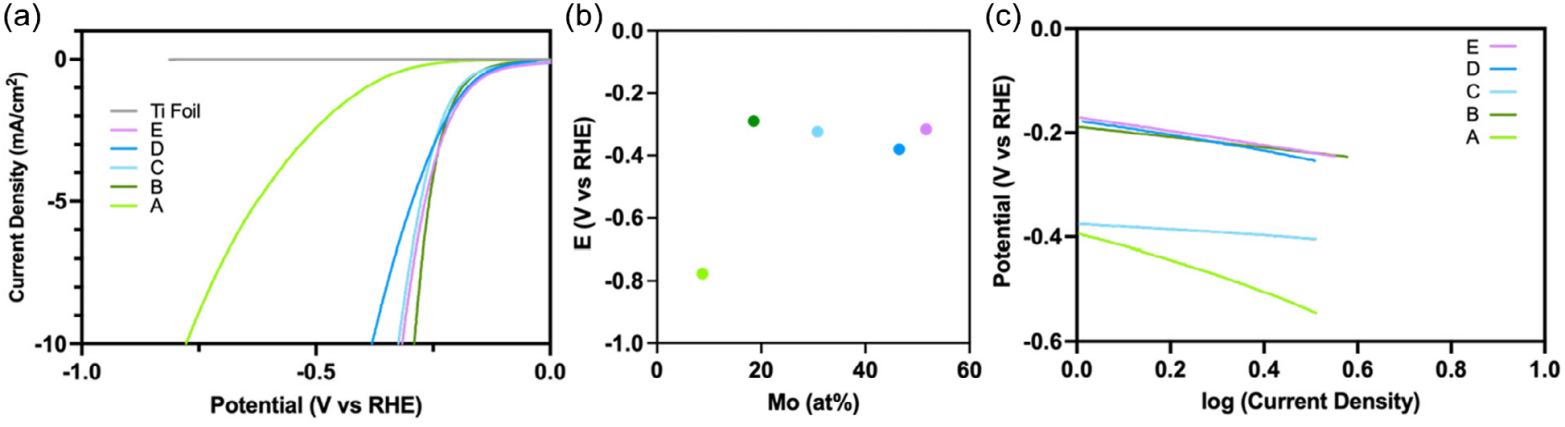
(a) Polarization curves of samples *A–* *E* in a pH 1.3 0.1 M NaClO_4_ solution. (b) Trend in the catalytic overpotentials obtained from the polarization plots. (c) Tafel plots derived from the polarization plots for alloy compositions *A–* *E*.

Polarization curves obtained from LSV were used to evaluate the electrocatalytic HER activity of the NiMo alloy nanoparticles (Figure 3a). The onset potential, defined as the potential required to reach a current density of 0.5 mA cm^−2^, was −333, −157, −158, −140, and −129 mV versus RHE for alloys *A*–*E*, respectively. These values indicate a strong composition dependence, with catalytic onset shifting more positively as the Ni:Mo ratio decreased. The alloy with the highest Mo content (alloy *E*) exhibited the lowest onset potential (−129 mV vs. RHE). The overpotential required to reach a current density of 10 mA cm^−2^ (*η*_10_) was next compared across the compositional series (Figure 3b). Alloy *B* (nominal Ni:Mo = 1:1) exhibited the lowest *η*_10_ of −290 mV versus RHE, while alloys *C*, *D*, and *E* gave *η*_10_ values of −324, −380, and −316 mV versus RHE, respectively. Alloy *A* displayed the highest *η*_10_ of −777 mV versus RHE. These results reveal that the HER activity is also composition‐dependent, with the overpotential minimized near nominal equimolar Ni:Mo ratios and slightly increasing for both Ni‐ and Mo‐rich compositions. Interestingly, the trend in onset potential does not mirror that of the overpotential, suggesting that the Ni:Mo ratio influences the reaction kinetics and active site distribution differently across the compositional series. To ensure that the observed HER activity originated from the NiMo alloy nanoparticles rather than the bare Ti substrate, a control experiment using a bare annealed Ti electrode was performed under identical conditions. The corresponding polarization curve showed negligible current, reaching only 10 µA cm^−2^ at the end of the potential window (Figure 3a), confirming minimal background HER activity from Ti.

The Tafel plots derived from the polarization curves were used to evaluate the HER kinetics of the five alloy compositions (Figure 3c). Among them, alloy *C* exhibited the lowest Tafel slope of 58 mV dec^−1^, indicating that the Volmer (hydrogen atom adsorption) step was not rate‐determining. In contrast, alloys *A*, *B*, *D*, and *E* gave Tafel slopes of 300, 104, 152, and 136 mV dec^−1^, respectively. These results reveal a clear composition‐dependent trend, suggesting that the Ni:Mo ratio strongly influences both the overpotentials and mechanistic pathway of the HER. The optimal performance was observed for alloy *B* due to its lowest overpotential. Alloy *B* was synthesized from a nominal 1:1 Ni:Mo precursor ratio, corresponding to an experimentally determined composition of Ni_0.86_Mo_0.14_. This alloy (hereafter referred to as Ni_0.86_Mo_0.14_) was selected for further investigation of hydrogen production selectivity. Controlled‐potential electrolysis (CPE) conducted at −0.368 V versus RHE (slightly above *η*_10_) produced a steady current density of ∼15 mA cm^−2^ over 2 h, with a Faradaic efficiency (FE) exceeding 99% for hydrogen evolution (Figure S7a). A double‐layer capacitance (*C*_dl_) value of 44 µF was obtained (Figure S8), which corresponded to an electrochemically active surface area (ECSA) value of 1.26 cm^2^. A long‐term CPE experiment at an applied potential of −290 mV versus RHE, corresponding to a geometric current density of 10 mA cm^−2^ for 45 h showed current response such that the material was activated in the first ∼5 h reaching a current density of ∼30 mA cm^−2^ (Figure S7b). The current density was maintained until ∼20 h, after which the current decreased to about ∼20 mA cm^−2^ toward the end of the experiment, possibly due to depletion of the proton substrate. Post‐catalysis XPS confirmed the presence of both Ni and Mo after 45 h of CPE (Figure S9, Supporting Information).

### 3.3 Recycling the IL Solvent

IL solvents have been used and successfully recycled and reused in the synthesis of nanoparticles [10, 11, 32, 33, 34]. However, these previous studies have not demonstrated whether the functionality of the resulting nanoparticles is maintained upon solvent recycling. Here, the BMIM‐NTf_2_ solvent was recovered after the synthesis and isolation of the NiMo nanoparticles. The recovered BMIM‐NTf_2_ appeared opaque dark brown, compared to the clear light‐yellow color of the virgin IL, suggesting the presence of dissolved metal species. Because elemental analysis showed that the NiMo alloy nanoparticles contained less Mo than the synthetic feed ratio, and because it served as a reducing agent, the dissolved metal content was assumed to consist mainly of oxidized Mo species. To purify the recovered IL for reuse, an aqueous extraction was performed. Previous studies used acidic aqueous solutions to strip residual metal ions from ILs [8, 9, 11]; however, in the present study, extraction with acidic solutions did not return the BMIM‐NTf_2_ to its clear light‐yellow color, indicating incomplete removal of metal ions. Consequently, basic extraction media were explored, as previous reports have shown that oxidized Mo species preferentially partition into the basic aqueous phase when imidazolium‐based ILs are used [35]. Initial trials with NaOH solutions successfully removed the color but left behind persistent NaOH residues after conventional aqueous workup. Replacing NaOH with NH_4_OH proved more effective, as its volatility allowed for its complete removal under vacuum, yielding purified, light‐yellow BMIM‐NTf_2_ suitable for reuse.

In the recycling campaign reported here, we performed an initial synthesis using virgin BMIM‐NTf_2_ followed by five successive syntheses using the recovered IL (i.e., five recycling cycles; six syntheses in total). The final catalyst denoted Ni_0.86_Mo_0.14_‐R was prepared using BMIM‐NTf_2_ that had been recycled five times. To begin the recycling process, the best performing alloy, Ni_0.86_Mo_0.14_, was first synthesized using virgin BMIM‐NTf_2_, after which the nanoparticles and IL were isolated. The recovered IL phase was then extracted with aqueous NH_4_OH, worked up, and dried. This, and each subsequent purification cycle, gave an average IL recovery yield of 66% (Table S2), which could likely be improved by scaling up the overall reaction [10]. An appropriate amount of virgin BMIM‐NTf_2_ was added to the recovered IL to maintain a constant solvent volume for subsequent syntheses. The nanoparticle synthesis, IL recovery, purification, and reuse was repeated for a total of six syntheses, requiring replenishment of ca. 34% of the solvent volume for each reaction after the first synthesis. Thus, over six cycles of nanoparticle synthesis, a total of 2.7 volume equivalents of IL solvent were required, as compared to 6 volume equivalents in the traditional organic solvent‐based route (vide infra).

**FIGURE 4:**
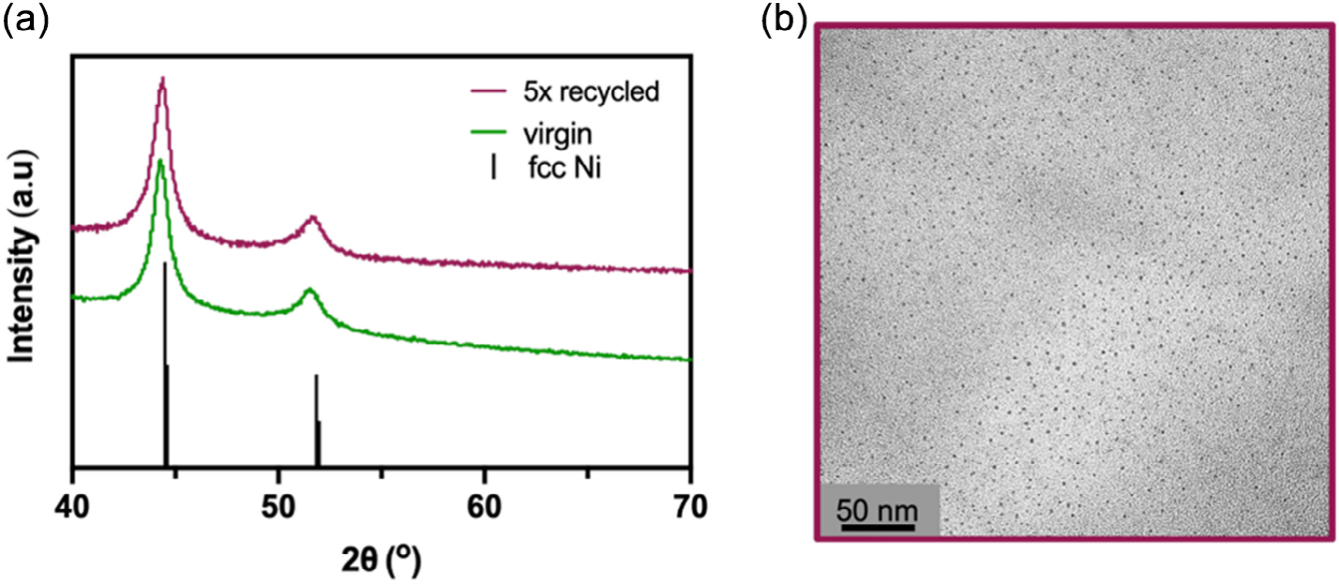
(a) Powder XRD diffraction pattern of Ni_0.86_Mo_0.14_ nanoparticles synthesized in virgin BMIM‐NTf_2_ (green) and 5x recycled BMIM‐NTf_2_ (red) with the corresponding stick pattern for FCC Ni (black). (b) TEM image of Ni_0.86_Mo_0.14_‐R nanoparticles synthesized in 5x recycled BMIM‐NTf_2_.

**FIGURE 5:**
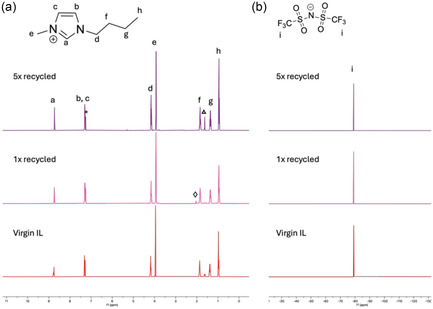
(a) ^1^H NMR spectrum of virgin, 1x, and 5x recycled BMIM‐NTf_2_ IL with the structure of the cation and the corresponding proton peaks labeled both in the structure and the spectra. (b) ^19^F NMR spectrum of virgin, 1x, and 5x recycled BMIM‐NTf_2_ with the structure of the anion and corresponding F peak labeled. The residual solvent peak at 7.26 ppm from CDCl_3_ is labeled with an asterisk (*). Residual water at 1.56 ppm is labeled with a triangle (▵) and residual acetone from IL recycling at 2.17 ppm is labeled with a diamond (⋄) in the ^1^H spectrum.

The nanoparticles obtained using the five‐times recycled BMIM‐NTf_2_ are denoted Ni_0.86_Mo_0.14_‐R. Figure 4a shows the powder XRD patterns of Ni_0.86_Mo_0.14_ nanoparticles synthesized in virgin BMIM‐NTf_2_ and Ni_0.86_Mo_0.14_‐R nanoparticles synthesized in five‐times recycled BMIM‐NTf_2_. Both samples yielded phase‐pure NiMo alloy nanoparticles, with no differences in the positions or breadths of the diffraction peaks, indicating that IL recycling had no effect on nanoparticle crystallinity. TEM analysis also revealed that the particle size (3.6 ± 0.6 nm) and morphology remained consistent when using recycled versus virgin IL (Figure 4b). To further assess the stability of the IL through the recycling process, solution ^1^H and ^19^F NMR spectra were collected after each recovery and purification cycle (Figure 5). Across all recycling cycles the spectra showed no emergence of new resonances, peak broadening, or chemical shift changes relative to virgin BMIM‐NTf_2_. These observations indicate that neither cation degradation nor anion decomposition occurs under the applied washing conditions or repeated heating, confirming the chemical stability of BMIM‐NTf_2_ under the applied recycling conditions. A representative ^1^H NMR spectrum of unwashed IL, compared with virgin and washed samples, showed broadening of the most downfield proton resonance in the presence of the dissolved metal content (Figure S10); however, no such peak broadening was observed for the recycled IL, further confirming the successful removal of residual metal ions. The number of cycles evaluated was limited by the practicality of manual batch recycling rather than by evidence of solvent degradation. Consistent with prior reports from our group demonstrating the structural stability of BMIM‐NTf_2_ and related ILs over multiple nanoparticle synthesis cycles [10, 11, 36], these results suggest robust chemical stability under the applied reaction and purification conditions. With an average recovery of 66% per cycle, the fraction of original solvent remaining follows (0.66)^*n*^, which indicates that after approximately 10 recycling cycles, less than 2% of the initially charged IL would remain. Thus, under the current recovery efficiency, the solvent inventory is effectively renewed on this timescale even in the absence of chemical degradation.

As a further comparison, Ni_0.86_Mo_0.14_ nanoparticles were synthesized in a mixture of oleylamine and octadecene, which are the typical organic nanocrystal synthesis solvents [23]. After one synthesis, the disappearance of the peak corresponding to the *α* hydrogens of oleylamine (*t*, 2.6 ppm) was observed in the ^1^H NMR spectrum as well as the emergence of a new peak at 3.26 ppm that most likely corresponds to aldimine, a known oleylamine decomposition product (Figure S11) [37]. We note that the aqueous NH_4_OH wash volume is small and quantified in the Experimental section (10 mL NH_4_OH + 2 mL water per purification cycle, relative to 2.5 mL IL charge). Over the six syntheses reported here, this corresponds to 60 mL of aqueous waste (5 purifications) in addition to 4.25 mL of unrecoverable IL, compared to 15 mL of discarded organic solvents in a conventional route (6 full solvent replacements). Thus, while the IL route produces a larger volume of aqueous waste, it reduces total consumption of organic VOCs and the mass of hazardous organic solvent waste requiring disposal, and the aqueous stream can be neutralized and treated by standard wastewater procedures.

ICP‐OES was carried out on the five‐times recycled IL to quantify the extent of Mo stripping achieved with aqueous NH_4_OH extraction. For one synthesis of Ni_0.86_Mo_0.14_, it can be estimated that 25% of the added Mo(CO)_6_ is incorporated into the alloy nanoparticles, leaving up to 75% of the Mo species dissolved in the IL phase. This corresponds to roughly 0.3 mmol of Mo remaining in the 2.5 mL reaction volume, or a maximum of 11,520 ppm prior to washing. ICP‐OES analysis of BMIM‐NTf_2_ following six syntheses and five recycling cycles detected only 132 ppm of Mo, which is equivalent to approximately 2.75 μmol of Mo in a 2 mL sample of IL (example volume recovered after recycling). Less than 8.7 ppm of Ni was detected by ICP‐OES as well, confirming effective removal of residual metal species from the recycled IL.

**FIGURE 6:**
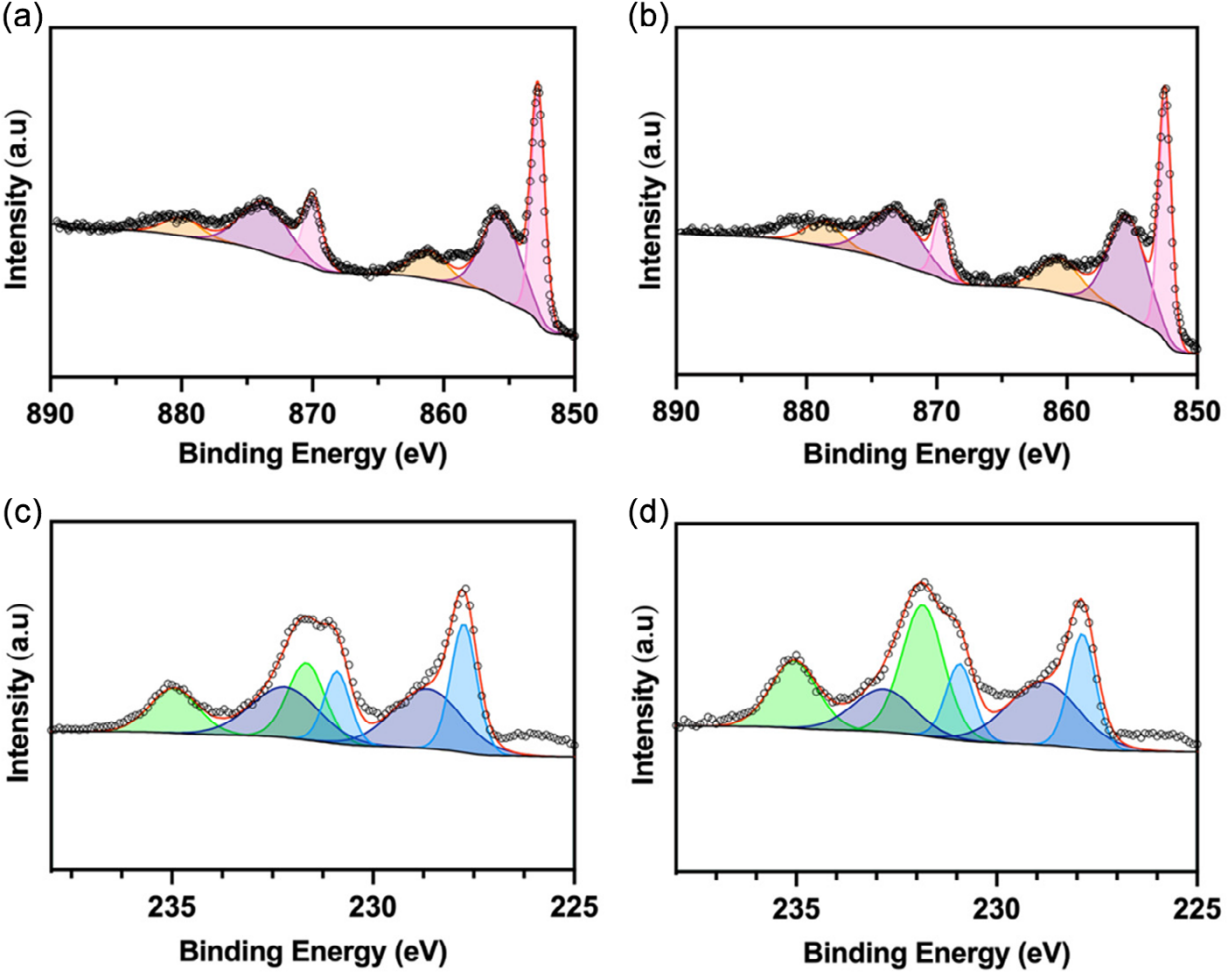
High‐resolution XPS spectra of Ni_0.86_Mo_0.14_ synthesized in virgin IL in the (a) Ni 2*p* and (c) Mo 3*d* core level regions. High‐resolution XPS spectra of Ni_0.86_Mo_0.14_‐R in the (b) Ni 2*p* and (d) Mo *3d* core level regions. There is no appreciable difference in binding states present between the electrode made in virgin versus recycled IL.

Prior to electrochemical testing, electrodes prepared from Ni_0.86_Mo_0.14_ synthesized in virgin IL and from Ni_0.86_Mo_0.14_‐R revealed no differences in the Ni 2*p* and Mo 3*d* binding environments (Figure 6). The high‐resolution Ni 2*p* spectra show zero‐valent Ni^0^ 2*p* peaks at binding energies of 852.8 and 870.0 eV, Ni^2+^ peaks at binding energies of 855.6 and 873.7 eV, and satellite peaks at 861.1 and 880.0 eV (Figure 6a,b). The Mo 3*d* spectra show zero‐valent Mo^0^ 3*d* peaks at binding energies of 227.8 and 230.8 eV, as well as Mo^6+^ peaks at 231.8 and 235.0 eV, with additional features at 228.8 and 232.7 eV attributed to Mo^3+^, consistent with previous reports (Figure 6c,d) [17, 23, 38].

### 3.4 HER Activity of the NiMo Nanoparticles Synthesized Using Recycled IL

**FIGURE 7:**
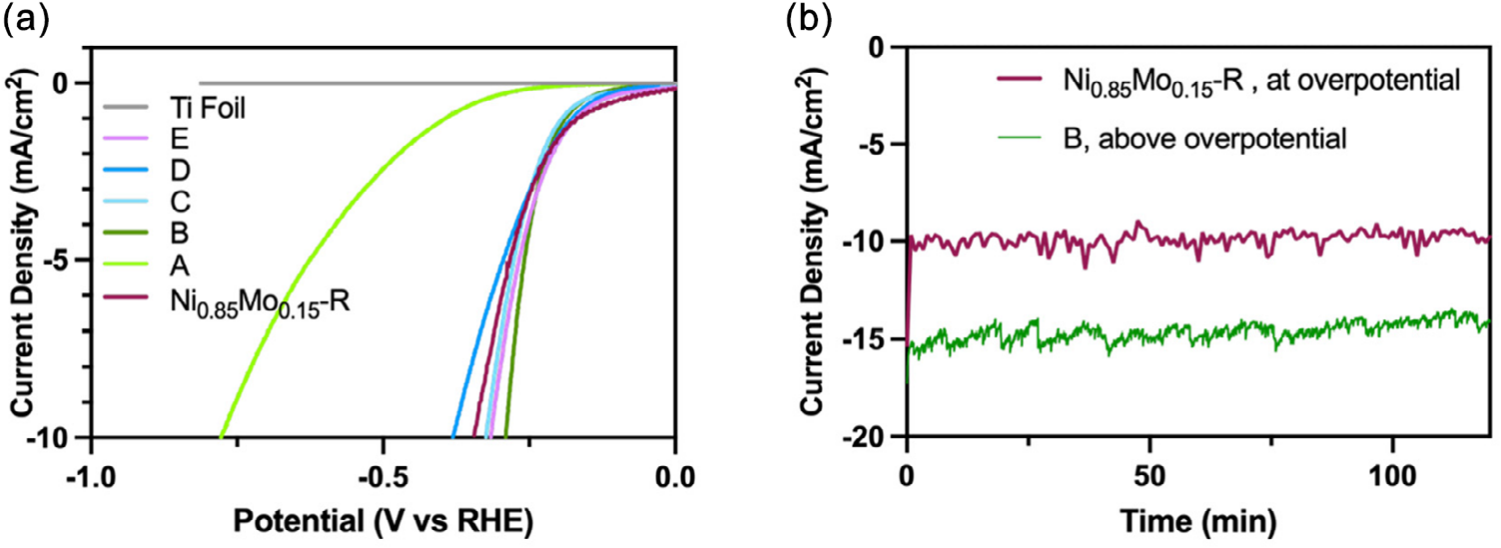
(a) Polarization plots of alloys *A–* *E* compared with Ni_0.86_Mo_0.14_‐R in a pH 1.3 0.1 M NaClO_4_ solution, and (b) 2 h CPE trace of alloy B (Ni_0.86_Mo_0.14_) and Ni_0.86_Mo_0.14_‐R above and at the overpotential, respectively, showing a stable current response.

The Ni_0.86_Mo_0.14_‐R nanoparticles were drop‐cast onto Ti electrodes and annealed in the same manner as the catalysts synthesized in virgin IL to study the effect of IL recycling on HER performance. LSV revealed an onset potential of −135 mV versus RHE and an overpotential at 10 mA cm^−2^ (*η*_10_) of −351 mV versus RHE (Figure 7a). For comparison, the Ni_0.86_Mo_0.14_ nanoparticles prepared in virgin IL exhibited an onset potential of −157 mV and an *η*_10_ of −290 mV versus RHE. Although the onset improved slightly for the sample synthesized in recycled IL, the higher *η*_10_ indicates slower apparent kinetics when evaluated on a geometric basis. Consistent with this observation, Tafel analysis yielded a slope of 205 mV dec^−1^ for Ni_0.86_Mo_0.14_‐R, compared to 104 mV dec^−1^ for the Ni_0.86_Mo_0.14_ electrocatalyst (Figure S12). To determine whether this difference reflects a change in intrinsic activity or simply variations in accessible surface area, double‐layer capacitance measurements were performed. The Ni_0.86_Mo_0.14_‐R nanoparticles exhibited a *C*_dl_ value of 15 µF, corresponding to an ECSA of 0.43 cm^2^ (Figure S13), whereas Ni_0.86_Mo_0.14_ synthesized in virgin IL gave an ECSA of 1.26 cm^2^.

Although XRD and TEM confirm that the nanoparticles synthesized in virgin and recycled IL are indistinguishable in terms of crystallinity, phase, and primary particle morphology, ECSA reflects the electrolyte‐accessible surface area of the deposited electrode film rather than the intrinsic structure and morphology of the nanoparticles. ECSA is inherently sensitive to mesoscale variations in film morphology and electrolyte accessibility, particularly for electrodes prepared by drop‐casting from colloidal suspensions. Such differences can influence the accessible surface area without altering nanoparticle size or composition. When polarization curves were normalized to ECSA rather than geometric surface area, both catalysts displayed similar current densities (Figure S14), indicating that IL recycling does not significantly alter the intrinsic HER activity of the catalysts. The slightly lower ECSA of Ni_0.86_Mo_0.14_‐R therefore accounts for the ∼60 mV increase in geometric overpotential.

Catalyst stability was first evaluated by CPE at the applied overpotential for 2 h (Figure 7b), which showed a stable current response with a FE > 99% for hydrogen evolution. Long‐term stability of Ni_0.86_Mo_0.14_‐R was subsequently assessed by a 45 h CPE experiment at an applied potential of −351 mV versus RHE, corresponding to a current density of 10 mA cm^−2^ (Figure S15). Both Ni_0.86_Mo_0.14_ and Ni_0.86_Mo_0.14_‐R exhibited similar current profiles throughout the extended electrolysis period, including an initial activation phase followed by sustained activity. Postelectrolysis XPS analysis confirmed the continued presence of both Ni and Mo after 45 h of operation (Figures S9 and S16). In both the Ni_0.86_Mo_0.14_ and Ni_0.86_Mo_0.14_‐R electrodes, loss of Ni peak resolution and the presence of Mo^6+^ features (i.e., at binding energies of 231.8 and 235.0 eV) were observed after prolonged electrolysis, indicating Ni loss and surface oxidation under acidic HER conditions [39]. In alkaline environments, Mo leaching from the electrode can create Ni‐rich surfaces [40, 41], though post‐catalytic analysis of metal leaching for HER in acidic conditions has been limited [16, 17]. Previous studies have also shown that NiMo catalysts experience activity degradation during prolonged water splitting [42]. Importantly, these changes were similar for catalysts synthesized in virgin and recycled IL, suggesting they are intrinsic to the material and arise from extended operation in acidic media rather than changes induced by the solvent recycling process.

## 4 Conclusion

In summary, we report a rapid microwave‐assisted synthesis of NiMo alloy nanoparticles in the ionic liquid BMIM‐NTf_2_, integrated with a systematic evaluation of solvent recovery and reuse. An initial synthesis in virgin IL followed by five successive recycling cycles, corresponding to six total syntheses, demonstrates that BMIM‐NTf_2_ can serve as both reaction medium and recyclable solvent while maintaining nanoparticle integrity. Quantitative solvent accounting shows that aqueous NH_4_OH purification removes approximately 99% of dissolved Mo species and yields an average IL recovery of 66% per cycle, resulting in a total requirement of 2.7 volume equivalents of IL across the six syntheses reported here, compared to complete solvent replacement in conventional organic routes.

Nanoparticles synthesized in five‐times recycled IL exhibit crystallinity, morphology, and intrinsic HER activity comparable to those prepared in pristine solvent. Although modest differences in geometric overpotential are observed, ECSA‐normalized polarization curves indicate that these differences arise from variations in accessible surface area rather than changes in intrinsic catalytic activity. Extended 45 h electrolysis experiments further demonstrate comparable operational stability for catalysts prepared in virgin and recycled IL. Postelectrolysis structural and compositional analyses reveal that catalyst degradation under prolonged acidic cycling originates from Ni leaching and surface oxidation inherent to HER conditions, rather than from the solvent recycling procedure.

Together, these findings establish that BMIM‐NTf_2_ can be efficiently recovered and reused in a closed‐loop process without compromising nanoparticle structure or catalytic performance. More broadly, this work demonstrates that solvent lifecycle considerations can be incorporated into nanoparticle electrocatalyst synthesis, providing a sustainability‐oriented framework that reduces solvent waste while preserving functional materials properties.

## Supporting Information

Additional supporting information can be found online in the Supporting Information section.

## Funding

This study was supported by U.S. Department of Energy (DE‐FG02‐11ER46826, DE‐SC0019236).

## Conflicts of Interest

The authors declare no conflicts of interest.

## Supporting information

Supplementary Material

## Data Availability

The data that support the findings of this study are available from the corresponding author upon reasonable request.
