# Supplementary material for: Sustainable Synthesis of NiMo Alloy Nanoparticles for Hydrogen Evolution Catalysis Using a Recyclable Ionic Liquid Solvent
Source: ChemSusChem. 2026 Apr 13;19(7):e70620. doi: 10.1002/cssc.70620 (PMC13076057; doi:10.1002/cssc.70620)
Supplement: Supplementary file 1 — Supplementary Material [file CSSC-19-e70620-s001.pdf]

# Supporting Information

## Sustainable Synthesis of NiMo Alloy Nanoparticles for Hydrogen Evolution Catalysis Using a Recyclable Ionic Liquid Solvent

Allison P. Forsberg[a†], Yashna Khakre[a†], Smaranda Marinescu[a], and Richard L. Brutchey[a*]

[a]Department of Chemistry, University of Southern California, 840 Downey Way, Los Angeles, California 90089-0744, USA

[†]Authors contributed to this work equally

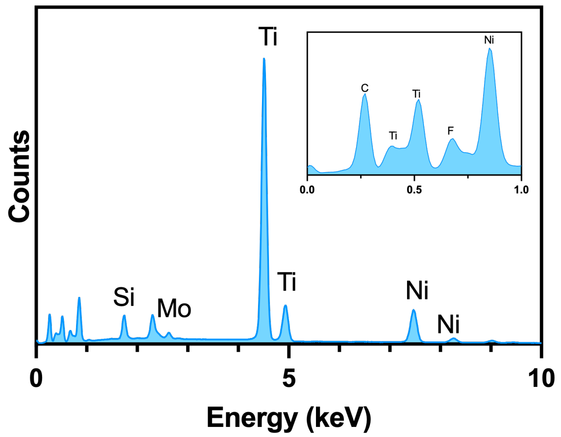


**Figure S1.** A SEM-EDX spectrum from the $Ni_{0.86}Mo_{0.14}$ electrode with the inset showing the spectrum from 0-1 keV. Si is from residual grease used to protect the borders of the active area to keep the dropcast nanoparticle suspension within the intended active area; F and C are from residual IL on the surface of the nanoparticles.

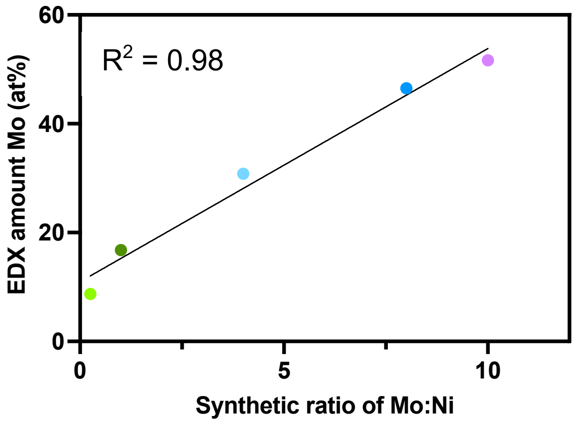


**Figure S2.** The nominal ratio of Mo:Ni versus the actual atomic percent of Mo detected by SEM-EDX. The slope of the linear regression line is 4, indicating that about 25% of the added amount of Mo is incorporated into the NiMo alloy nanoparticle.

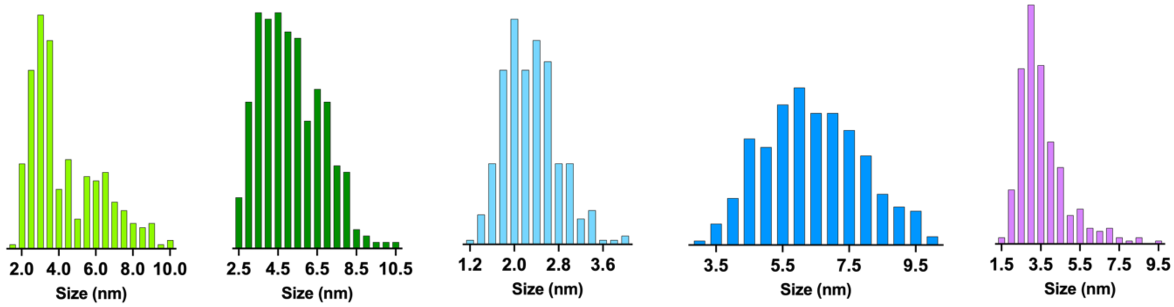


**Figure S3.** Nanoparticle size histograms of samples A-E. $N > 300$ particles were counted for each sample.

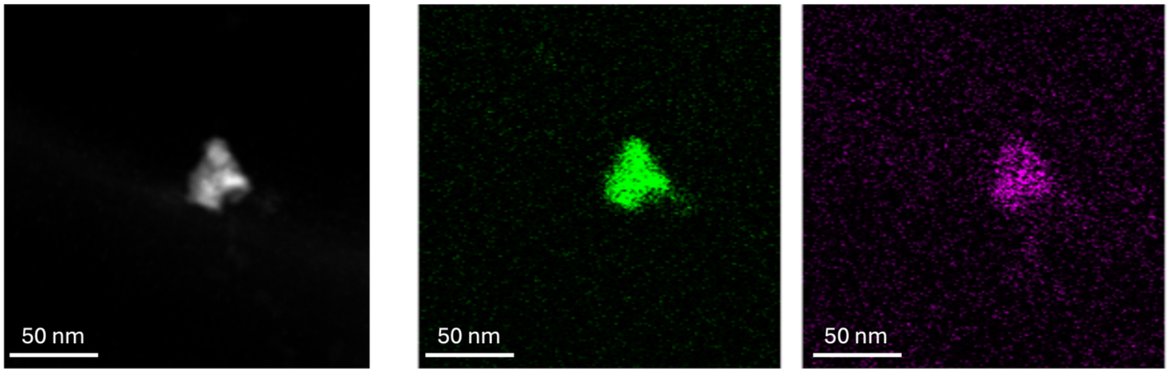


**Figure S4.** HAADF-STEM image of a cluster of $Ni_{0.85}Mo_{0.14}$ nanoparticles with the corresponding EDX maps for Ni (green) and Mo (purple). Note: Mo is present in the objective aperture, increasing the background noise.

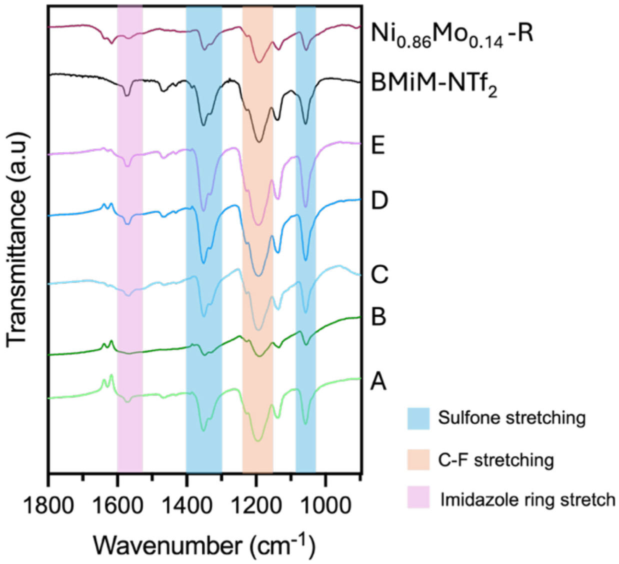


**Figure S5.** FT-IR spectra of all NiMo alloys (A-E), pure IL (BMiM-NTf$_2$), and Ni$_{0.86}$Mo$_{0.14}$-R. Sulfone stretching and C-F stretching arise from the anion while the imidazole ring stretch is from the cation of the IL.

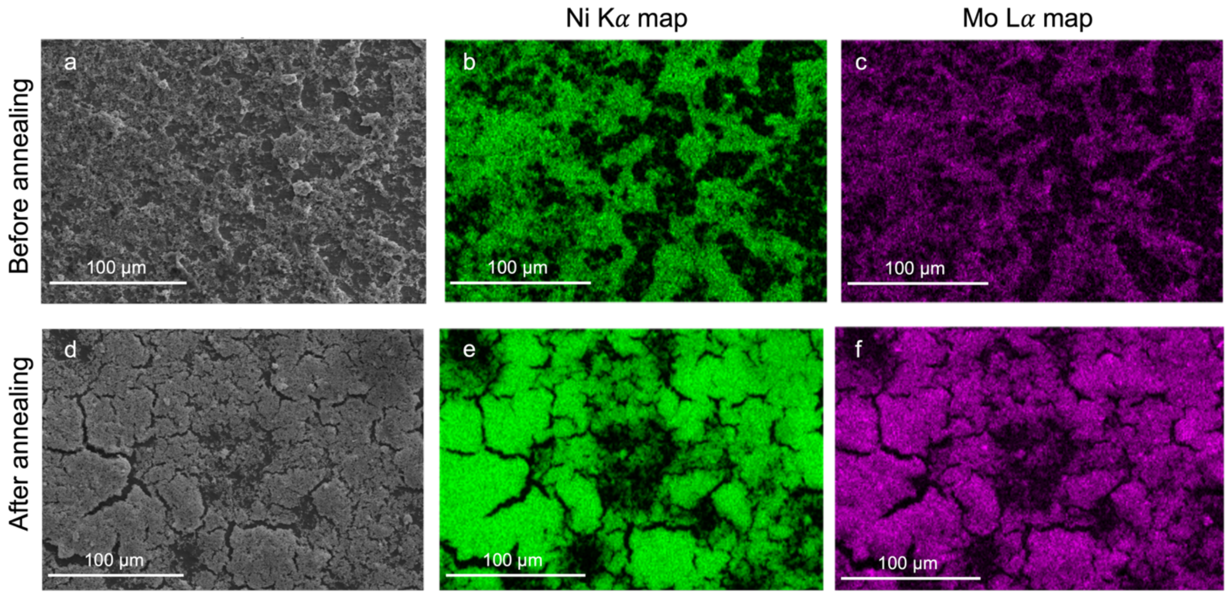


**Figure S6.** SEM images (a, d) and EDX maps of Ni *K*α (b, e) and Mo *L*α (c, f) of $Ni_{0.85}Mo_{0.15}$-R before annealing (a-c), after annealing under 5% $H_2$ at 450 ˚C for 1 h (d-f).

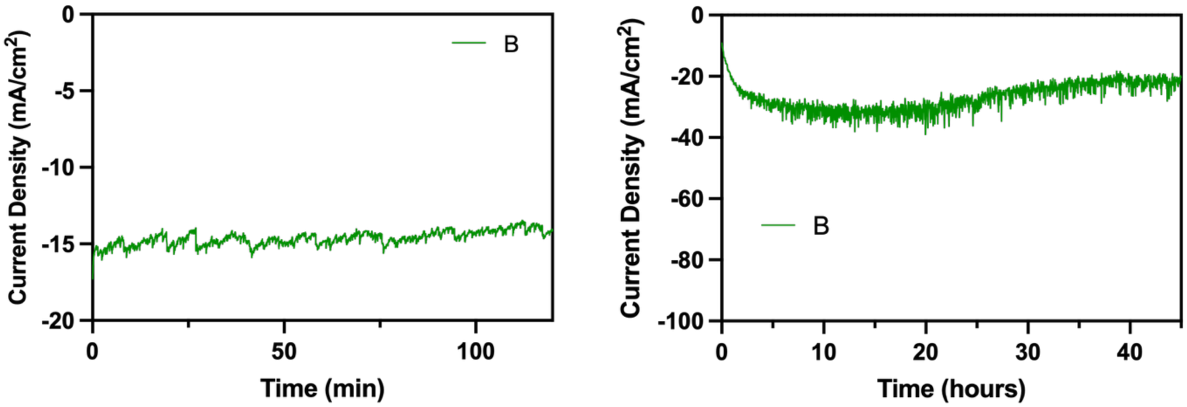


**Figure S7.** Controlled potential electrolysis trace of alloy B ($Ni_{0.85}Mo_{0.15}$) for (a) 120 min and (b) 45 h.

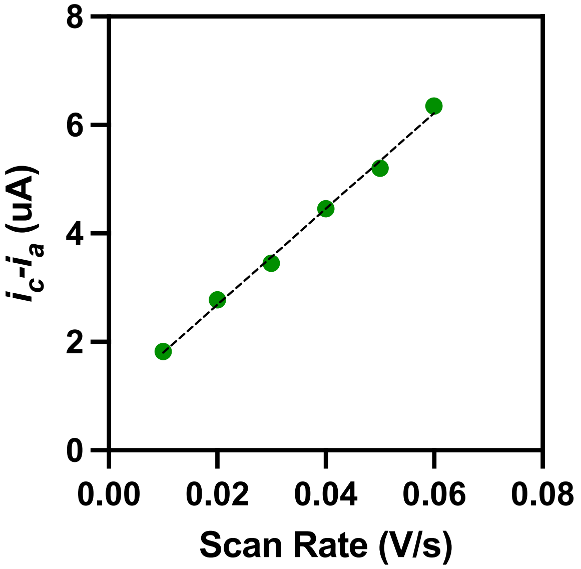


**Figure S8.** Double-layer capacitance measurement for alloy B ($Ni_{0.85}Mo_{0.15}$). The $C_{dl}$ was calculated to be 44 μF.

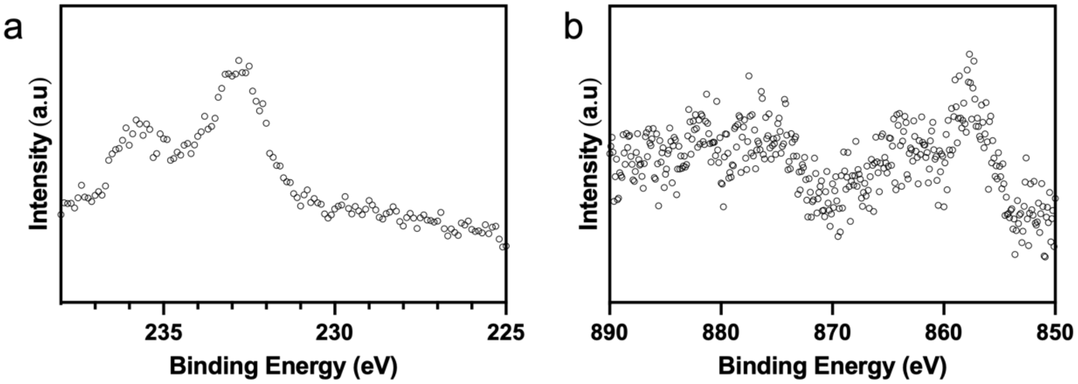


**Figure S9.** (a) Mo 3*d* and (b) Ni 2*p* XPS spectra of alloy B ($Ni_{0.85}Mo_{0.15}$) after 45 h of controlled potential electrolysis.

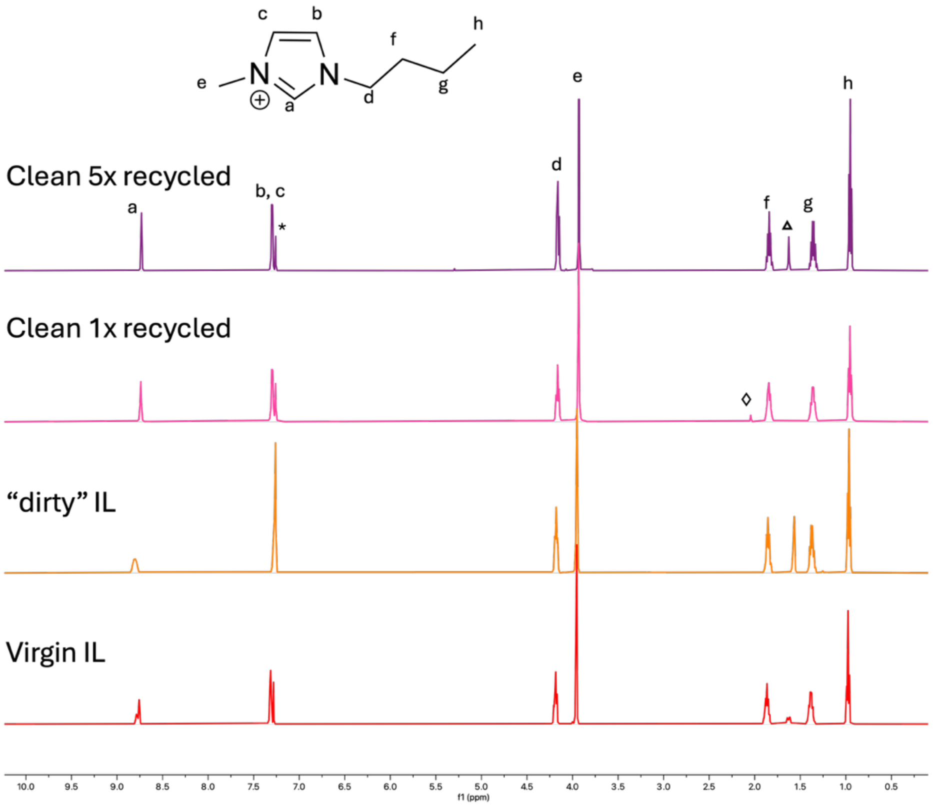


**Figure S10.** $^{1}$HNMR spectrum of the virgin IL (bottom, red), before extracting with $NH_4OH$, and after extracting and recycling 1× and 5×. The most downfield proton resonance, labeled a, showcases a broadening when the IL has not been treated with $NH_4OH$ to remove residual dissolved Mo ions. No change was observed in the $^{19}$F NMR spectrum prior to washing the IL when compared to the virgin IL. Residual water at 1.56 ppm is labeled with a triangle (▵) and residual acetone from IL recycling at 2.17 ppm is labeled with a diamond (◊) in the $^{1}$H spectrum.

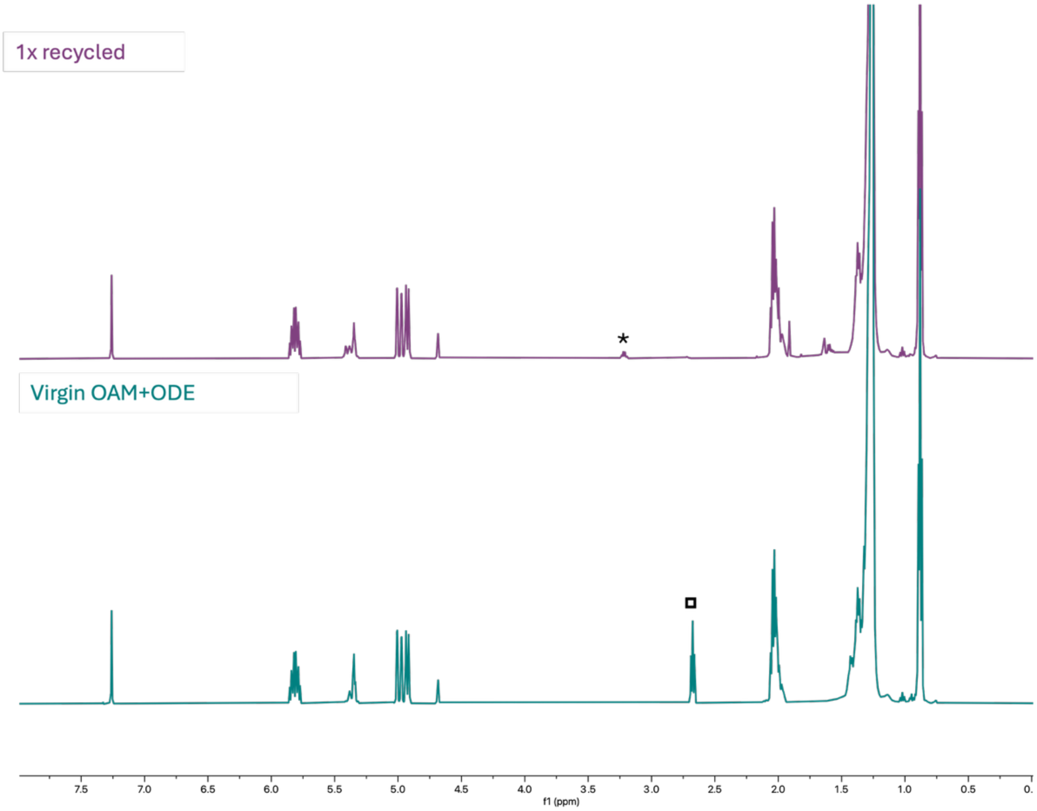


**Figure S11.** $^1$H NMR spectra of the octadecene (ODE) and oleylamine (OAm) mixture used to synthesize NiMo nanoparticles as previously reported[1] (bottom, blue) and $^1$H NMR of the same reaction solvent after one synthesis following the same MW synthesis conditions utilized here (top, purple). After one use, the resonance corresponding to the α hydrogens of OAm (*t*, 2.6 ppm, □) has disappeared and a new peak corresponding to an aldimine has appeared (3.26 ppm, *). Both spectra have been referenced to residual $CDCl_3$.

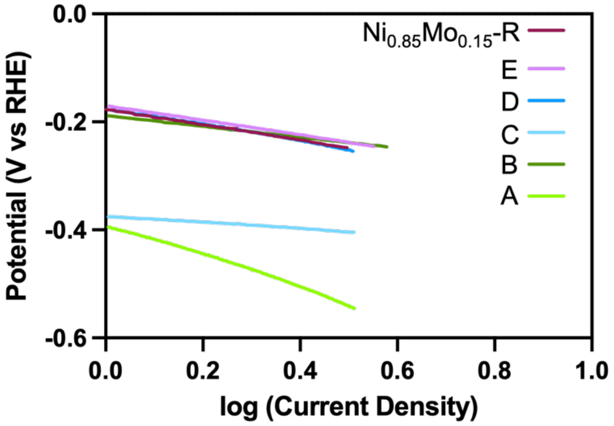


**Figure S12.** Tafel plot of $Ni_{0.85}Mo_{0.15}$-R compared with samples A-E synthesized using pristine BMIM-$NTf_2$ IL.

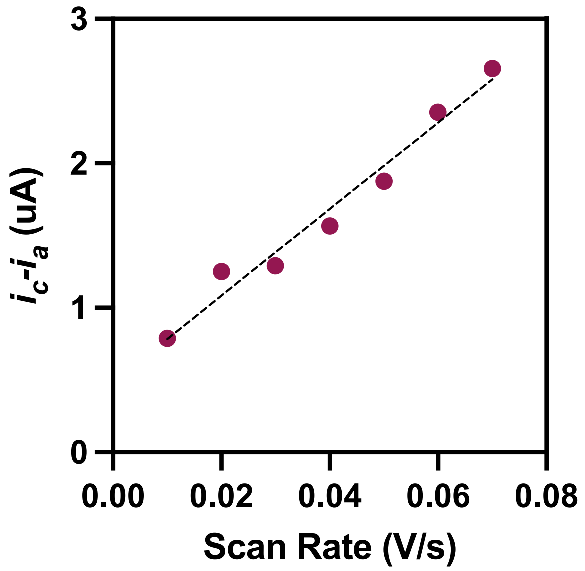


**Figure S13.** Double-layer capacitance measurement for $Ni_{0.85}Mo_{0.15}$-R. The $C_{dl}$ was calculated to be 15 μF.

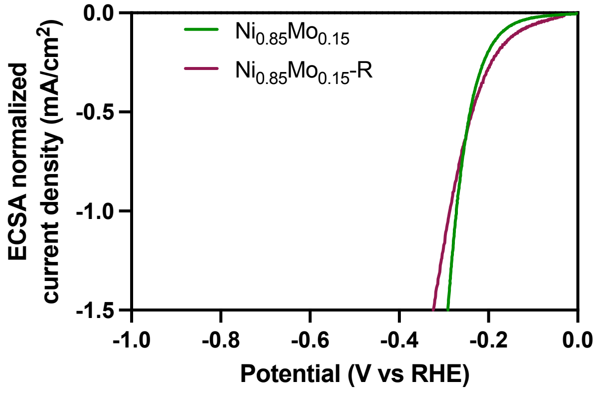


**Figure S14.** ECSA normalized polarization curves for $Ni_{0.85}Mo_{0.15}$ (green) synthesized in virgin BMIM-NTf$_2$ IL and $Ni_{0.85}Mo_{0.15}$-R (red) synthesized in 5× recycled BMIM-NTf$_2$ IL.

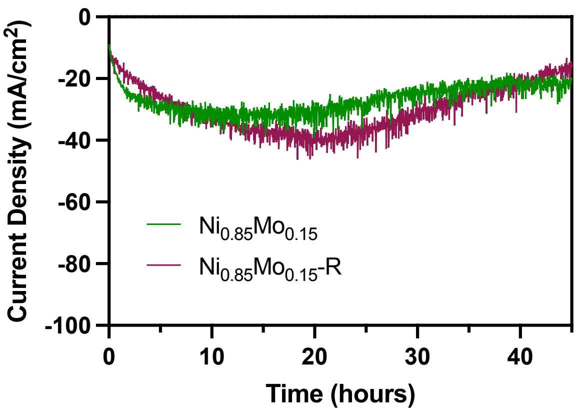


**Figure S15.** Controlled potential electrolysis traces for $Ni_{0.85}Mo_{0.15}$ and $Ni_{0.85}Mo_{0.15}$-R over 45 h.

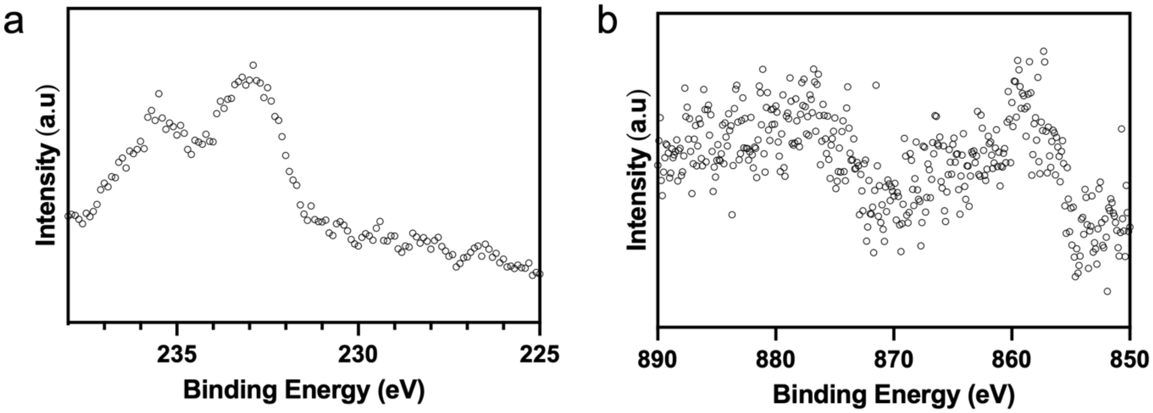


**Figure S16.** (a) Mo 3*d* and (b) Ni 2*p* XPS spectra of $Ni_{0.85}Mo_{0.15}$-R, after 45 h of controlled potential electrolysis.

**Table S1.** Elemental ratios of Ni and Mo in $Ni_{0.86}Mo_{0.14}$-R as determined by SEM-EDX at multiple points on each electrode.

| **Electrode sample** | **Ni atomic %** | **Mo atomic %** |
|---|---|---|
| Before annealing | 86.63 ± 0.16% | 13.37 ± 0.16% |
| After annealing | 86.53 ± 0.15% | 13.47 ± 0.15% |

**Table S2.** Volume of BMIM-$NTf_2$ recovered after each recycling and washing. The initial starting volume was 2.5 mL of virgin IL. Fresh IL was added to reach a total reaction volume of 2.5 mL for each subsequent reaction.

| 1$^{st}$ recovery | 2$^{nd}$ recovery | 3$^{rd}$ recovery | 4$^{th}$ recovery | 5$^{th}$ recovery |
|---|---|---|---|---|
| 1.3 mL | 1.8 mL | 1.9 mL | 1.6 mL | 1.6 mL |

**References:**


(1) Zhang, T.; Liu, X.; Cui, X.; Chen, M.; Liu, S.; Geng, B. Colloidal Synthesis of Mo–Ni Alloy Nanoparticles as Bifunctional Electrocatalysts for Efficient Overall Water Splitting. *Adv. Mater. Interfaces* **2018**, *5*, 1800359.

(2) Wan, C.; Regmi, Y. N.; Leonard, B. M. Multiple Phases of Molybdenum Carbide as Electrocatalysts for the Hydrogen Evolution Reaction. *Angew. Chem. Int. Ed.* **2014**, *53*, 6407–6410.

(3) *Handbook of X-Ray Photoelectron Spectroscopy: A Reference Book of Standard Spectra for Identification and Interpretation of XPS Data*, Update.; Moulder, J. F., Chastain, J., Eds.; Perkin-Elmer Corporation: Eden Prairie, Minn, 1992.
